# Immediate Interim Restoration Using Natural Tooth as a Pontic in Patients With Generalized Periodontitis: A Case Report

**DOI:** 10.7759/cureus.76805

**Published:** 2025-01-02

**Authors:** Mohd Nazar Rana, Enakshi Yadav, Manisha Rout, Mayur Kaushik

**Affiliations:** 1 Department of Periodontology and Implantology, Subharti Dental College and Hospital, Meerut, IND

**Keywords:** esthetic dentistry, esthetics dentistry, generalized periodontitis, natural tooth pontic, pontic, ribbond splinting

## Abstract

This case report describes an immediate interim restoration of hopeless teeth using natural teeth as pontics in patients diagnosed with generalized periodontitis. The loss of a tooth, particularly in the aesthetic zone, can significantly affect a patient's self-esteem and quality of life. In cases of generalized periodontitis, where tooth loss may be unavoidable, immediate replacement of the lost tooth is crucial for both aesthetic and functional reasons. A natural tooth used as a pontic is inherently the correct size, shape, and color, ensuring an aesthetically pleasing result without the need for extensive modification. This method leverages the existing contours and features of the patient's own tooth, providing a seamless transition in the smile. This case report explores the innovative use of a natural tooth as a pontic, bonded to adjacent abutment teeth using fiber-reinforced resin, offering a cost-effective and aesthetically pleasing solution.

## Introduction

The sudden loss of a tooth can be profoundly distressing for patients. Anterior teeth are critical not only for functional purposes, such as speech and mastication, but also for aesthetic reasons [1]. When a patient loses an anterior tooth - whether due to trauma, advanced periodontal disease, root resorption, or failed endodontic therapy - the emotional toll can be significant.

Given the emotional and functional implications of anterior tooth loss, many patients request immediate replacement options. Dentists are faced with the challenge of providing a solution that meets the aesthetic and functional needs of the patient while maintaining a favorable psychological state [2]. Although various permanent replacements, such as removable prostheses, tooth-supported prostheses, and implant-supported prostheses, are available, options for effective temporary prosthetics are more limited [3].

When acrylic removable partial dentures (RPDs) are placed immediately after tooth extraction, they can exert pressure on the healing tissues, potentially leading to discomfort and impaired healing. The rigid nature of these prostheses can also lead to irritation of the extraction site [4].

A natural tooth used as a pontic is inherently the correct size, shape, and color, ensuring an aesthetically pleasing result without the need for extensive modification. This method leverages the existing contours and features of the patient's own tooth, providing a seamless transition in the smile [5]. Furthermore, the use of a patient’s own natural tooth offers significant psychological advantages. Patients often feel more comfortable and confident when their restoration incorporates their own biological material, reinforcing a sense of normalcy and continuity [6]. The natural tooth that will serve as the pontic should be carefully extracted, ensuring minimal trauma to the surrounding tissues. The tooth's root surface is prepared to optimize bonding, usually involving cleaning and possibly etching. A plasma-treated woven polyethylene fiber, such as Ribbond, is used to reinforce the bonding process. This fiber enhances the strength and durability of the restoration [7].

## Case presentation

**Figure 1:**
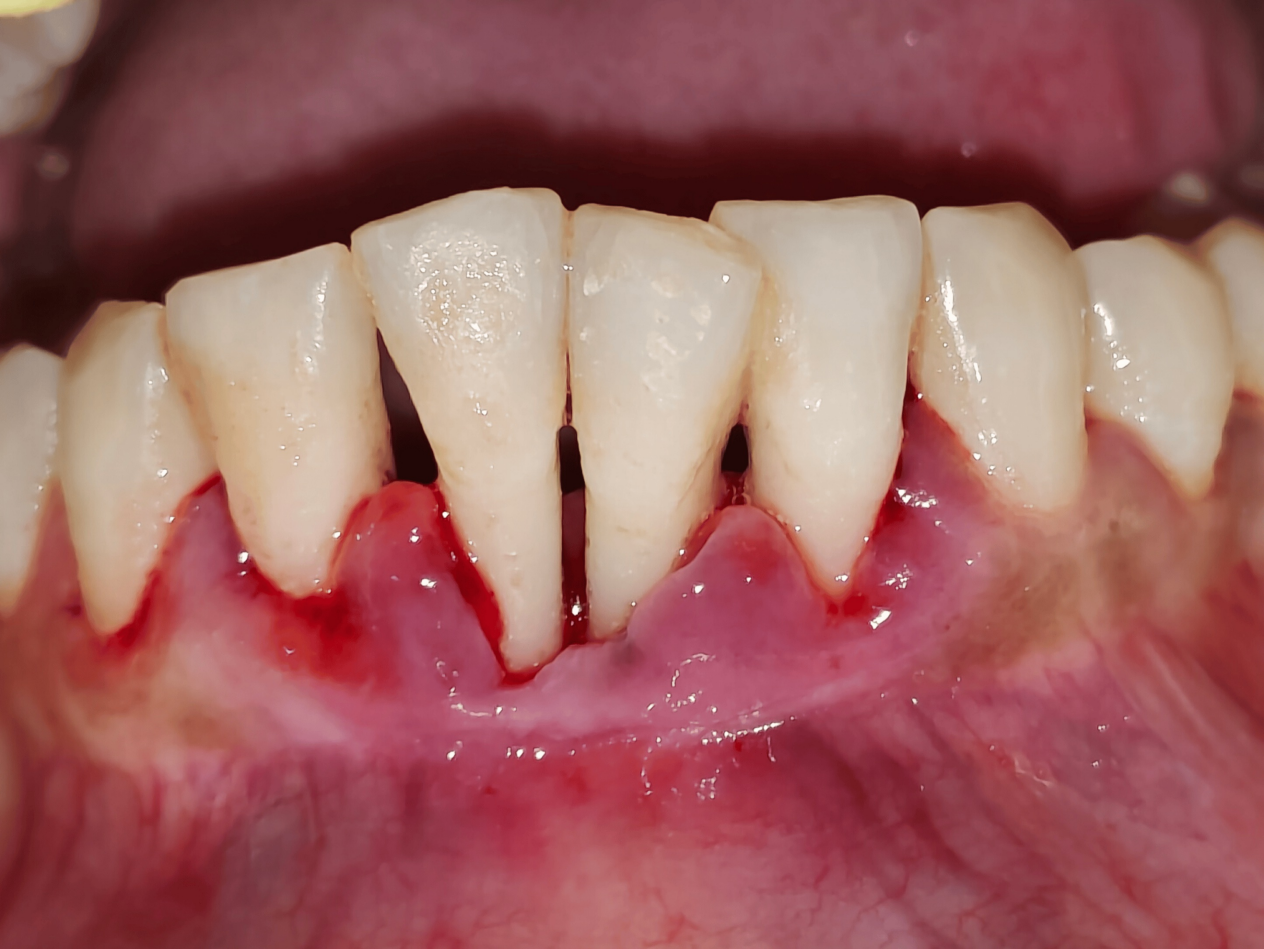
Preoperative view

A 36-year-old female non-smoker with an unremarkable medical history presented to the Department of Periodontology and Implantology complaining of severe mobility in her lower front tooth, which had persisted for one year (Figure 1).

**Figure 2:**
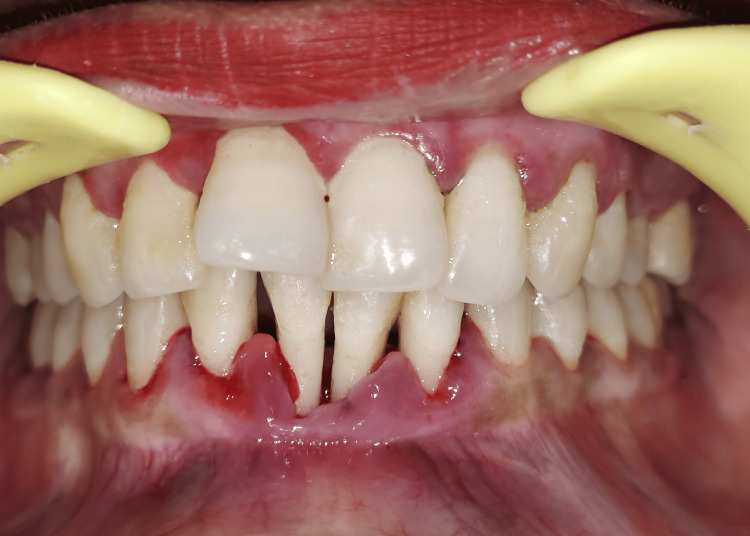
Preoperative view (in occlusion)

Clinical examination revealed generalized mobility (grade 1 and 2) and probing depths greater than 5 mm. After reviewing the patient’s history, clinical findings (gingival and periodontal condition, plaque, and calculus scores), and intraoral periapical (IOPA) radiographs, a diagnosis of generalized periodontitis was made (Figure 2).

The IOPA revealed significant bone loss around the mandibular right and left central incisor (#41 and #31), which exhibited grade 3 mobility. Clinical findings including periodontal probing depths of 10 mm on the distal and lingual surfaces and 8-9 mm on the mesial and facial surfaces of teeth #41 and #31 confirmed a hopeless prognosis, and extraction was planned. The right mandibular lateral incisor also showed grade 2 mobility and a 7-mm probing depth.

The patient, who had an important formal event to attend, requested immediate replacement of the extracted tooth and expressed interest in an implant for future restoration. Consequently, a single-visit, fiber ribbon-reinforced fixed partial denture was planned, using the crown of the extracted tooth as a natural tooth pontic, which would also function as a splint.

Full-mouth scaling and root planing were performed. Before extraction, the tooth's position in the arch and its relationship to the adjacent teeth were noted. Teeth #41 and #31 were extracted atraumatically without damaging the adjacent grade 2 mobile teeth, using rotational movement to avoid trauma. The natural tooth pontic’s length was determined by measuring from the incisal edge of the central incisor to the gingival margin of the extraction site. The root was separated from the crown using a straight fissure bur. The gingival side of the pontic was shaped and smoothed. The sectioned crowns were then assessed for size in the extraction site.

The enamel of the adjacent abutment teeth and the natural tooth pontics were etched on the lingual and proximal surfaces. A fiber ribbon was selected for the bridge-splint, given that all mandibular incisors were mobile due to periodontal disease. A piece of dental floss was used to measure the required length of the fiber ribbon, spanning from the mesial surface of the left mandibular canine to the mesial surface of the right mandibular canine. A bonding agent was applied to the etched teeth and fiber, followed by light curing. The fiber ribbon was placed along the lingual surfaces of the teeth and secured with flowable composite, which was then cured.

**Figure 3:**
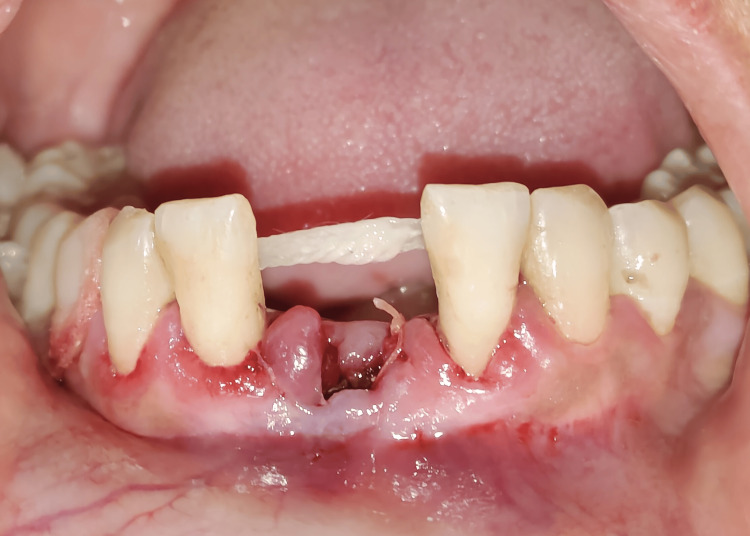
Splinting

**Figure 4:**
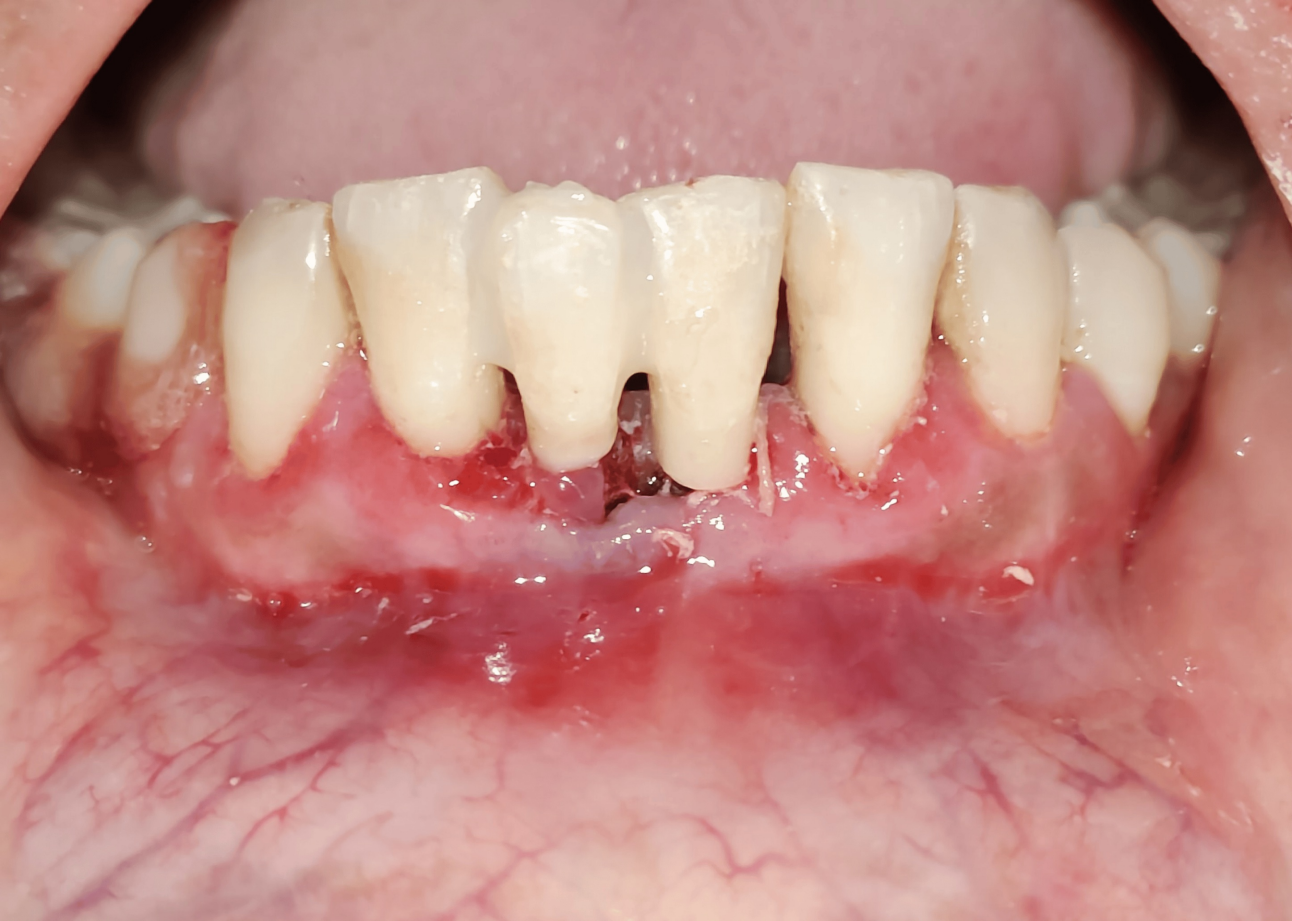
Splinting with natural teeth in place

The adjacent abutment teeth and pontic were bonded, and finishing and polishing were completed. Occlusal adjustments were made to ensure the pontic was not in contact with opposing teeth (Figures 3, 4).

The patient was instructed to use a proximal brush to clean the splinted area. A post-operative orthopantomogram confirmed no resin overhangs, and future flap and regenerative surgeries were planned for other areas based on the patient’s convenience.

The patient was educated on plaque control and underwent full-mouth periodontal flap surgeries with bone grafts as needed, along with appropriate antibiotics. Initially, the patient was seen monthly for three months after phase 2 therapy, followed by three-month intervals for two visits, and then six months. Oral hygiene instructions were reinforced at each visit, ensuring strict adherence to a hygiene regimen [2]. One year later, the patient remained highly satisfied with the aesthetics and function of the natural tooth pontic and had no desire to replace it.

Following the same methodology, two more patients were treated.

## Discussion

Using a natural tooth as a pontic offers numerous benefits, including aesthetic satisfaction, functional stability, and the potential to act as a splint for remaining teeth. This technique is particularly beneficial in patients with aggressive periodontitis, where immediate restoration is necessary to enhance both function and appearance. Tooth loss, particularly in the aesthetic zone, can lead to various complications, including shifting of adjacent teeth, midline deviation, space reduction, and over-eruption of opposing teeth [8]. Additionally, it may cause significant psychological distress and impair both speech and chewing. To temporarily address the space created by tooth extraction, options such as RPDs, resin-bonded fixed partial dentures, and, more recently, fiber-reinforced bridges can be used. However, studies indicate that fiber-reinforced bridges have a survival rate of only 28% over 7.5 years [9-10]. In this case, bonding was carried out using fiber-reinforced material, which is expected to improve the survival rate. Other factors such as the tooth’s position in the arch, chewing forces, and periodontal support of adjacent teeth may also influence the longevity of these pontics [11].

In the present case, the patient was more concerned for immediate restoration of lower anterior teeth, and thus interim prosthesis using natural teeth as pontics was the best option in this scenario. Given that several teeth exhibited some mobility, natural teeth pontics were used as a temporary solution [12]. It functioned as both a provisional restorative device and a splint while flap surgeries were performed. After completing both phase 1 and phase 2 treatments, the plan was to provide a more definitive restoration, such as a cast partial denture or implant-supported prosthesis [13]. However, the patient was not open to the idea of fixed partial dentures that required full-coverage crowns. One year later, the natural teeth pontics remain intact, both aesthetically and functionally, and the patient declined any further procedures at this time. In light of the patient’s wishes, further treatment has been postponed until future developments arise [14-15].

## Conclusions

The use of a natural tooth as a pontic represents a simple, cost-effective, and aesthetically pleasing solution for immediate tooth replacement in patients with generalized periodontitis. This case report illustrates the potential of this technique to provide effective interim restorations while maintaining the integrity of remaining dentition. Moreover, in situations where anterior teeth need to be removed, use of natural tooth pontics while the gingival tissue heals is an excellent and aesthetically acceptable treatment option and reflects the dentist’s concern for the patient’s aesthetic, functional, and psychological needs. Further studies with larger sample sizes and long-term follow-up are recommended to validate the effectiveness of this technique and to explore its applicability in different clinical scenarios.

## References

[1] Peterson SN, Snesrud E, Liu J, Ong AC, Kilian M, Schork NJ, Bretz W (2013). The dental plaque microbiome in health and disease. PLoS One.

[2] Craddock HL (2009). Consequences of tooth loss: 1. The patient perspective--aesthetic and functional implications. Dent Update.

[3] Stumpel LJ (2023). The natural tooth pontic; simplified. CDA J.

[4] Kretzschmar JL (2001). The natural tooth pontic: a temporary solution for a difficult esthetic situation. J Am Dent Assoc.

[5] Ashley M, Holden V (1998). An immediate adhesive bridge using the natural tooth. Br Dent J.

[6] Raj R, Mehrotra K, Narayan I, Gowda TM, Mehta DS (2016). Natural tooth pontic: an instant esthetic option for periodontally compromised teeth-a case series. Case Rep Dent.

[7] Kumar KP, Nujella SK, Gopal SS, Roy KK (2016). Immediate esthetic rehabilitation of periodontally compromised anterior tooth using natural tooth as pontic. Case Rep Dent.

[8] Wyatt CC (2007). Resin-bonded fixed partial dentures: what's new?. J Can Dent Assoc.

[9] Abdullai D, Akdemir O, Cengiz S, Cengiz MI (2023). Single tooth replacement using fiber-reinforced composite resin bridge - a case report. Int J Dent Res.

[10] Tuloglu N, Bayrak S, Tunc ES (2009). Different clinical applications of bondable reinforcement ribbond in pediatric dentistry. Eur J Dent.

[11] Saleem M, Tyagi A, Rana N, Kaushik M (2021). Root biomodification enhancing the predictability of isolated recession coverage-a 3 year follow-up case report. J Adv Med Dent Sci Res.

[12] Strassler H (2007). Single visit natural tooth pontic bridge with fiber reinforcement ribbon. Tex Dent J.

[13] Howe DF, Denehy GE (1977). Anterior fixed partial dentures utilizing the acid-etch technique and a cast metal framework. J Prosthet Dent.

[14] Rochette AL (1973). Attachment of a splint to enamel of lower anterior teeth. J Prosthet Dent.

[15] Tavangar MS, Aghaei F, Nowrouzi M (2022). Reconstruction of natural smile and splinting with natural tooth pontic fiber-reinforced composite bridge. Case Rep Dent.

